# Ising-like model replicating time-averaged spiking behaviour of in vitro neuronal networks

**DOI:** 10.1038/s41598-024-55922-9

**Published:** 2024-03-25

**Authors:** Cesar I. N. Sampaio Filho, Lucilla de Arcangelis, Hans J. Herrmann, Dietmar Plenz, Patrick Kells, Tiago Lins Ribeiro, José S. Andrade

**Affiliations:** 1 Departamento de Física, Universidade Federal do Ceará, Fortaleza, 60451-970 Brazil; 2 Department of Mathematics and Physics, University of Campania “Luigi Vanvitelli”, 81100 Caserta, Italy; 3 PMMH, ESPCI, CNRS UMR 7636, 7 Quai St. Bernard, 75005 Paris, France; 4 Section on Critical Brain Dynamics, NIMH, Bethesda, MD 20892 USA

**Keywords:** Phase transitions and critical phenomena, Computational neuroscience

## Abstract

We analyze time-averaged experimental data from in vitro activities of neuronal networks. Through a Pairwise Maximum-Entropy method, we identify through an inverse binary Ising-like model the local fields and interaction couplings which best reproduce the average activities of each neuron as well as the statistical correlations between the activities of each pair of neurons in the system. The specific information about the type of neurons is mainly stored in the local fields, while a symmetric distribution of interaction constants seems generic. Our findings demonstrate that, despite not being directly incorporated into the inference approach, the experimentally observed correlations among groups of three neurons are accurately captured by the derived Ising-like model. Within the context of the thermodynamic analogy inherent to the Ising-like models developed in this study, our findings additionally indicate that these models demonstrate characteristics of second-order phase transitions between ferromagnetic and paramagnetic states at temperatures above, but close to, unity. Considering that the operating temperature utilized in the Maximum-Entropy method is $T_{o}=1$, this observation further expands the thermodynamic conceptual parallelism postulated in this work for the manifestation of criticality in neuronal network behavior.

## Introduction

In 1985 Amit et al.^1^ for the first time drew a connection between neural networks and Ising spin glasses. Both systems have in common an energy landscape of many valleys and offer the possibility of delocalized storage of patterns. These analogies were explored further since then^2,3^. Subsequently, 1991 Miranda and Herrmann^4^ suggested that the brain operates at criticality in the sense that it exhibits avalanches of activity following power-law distributions for their size and duration, with exponents around − 1.5 and − 2.0, respectively. Alternative proposals were made later by Chialvo and Bak^5^. These theoretical predictions were experimentally confirmed by the seminal work of Beggs and Plenz^6^. By recording spontaneous local field potentials using a 60 channel multielectrode array on mature organotypic cultures of acute slices of rat cortex, they found power-law distributions in avalanche size and duration with similar exponents values as reported in^4,5^. Since then, many authors have confirmed signs of criticality in the brain^7–9^ overcoming the substantial challenges of studying scaling laws reported at criticality in the face of experimental constraints when assessing brain dynamics^10,11^.

**Figure 1:**
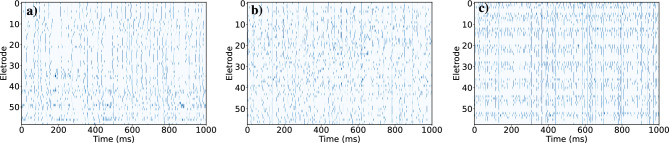
The ensemble of investigated time series of binarized spikes spanning a total of 1 s for 60 electrodes of the in vitro samples 1, 2 and 3 are shown in (**a**–**c**)^6^, respectively. Each horizontal line represents the temporal spiking series of a local group at an electrode or single neuron, respectively, whereas each vertical line represents the state of the system at time *t*. A blue point corresponds to a spike ($+\,1$) and an empty place to no spike ($-\,1$).

Models from Statistical Physics, when used in conjunction with the Maximum Entropy Method (MEM) developed in information theory, provide a conceptual framework to understand, from experimental data, a given natural process in terms of the “interactions” among its many elementary units^12–14^. The principle of maximum entropy, by itself, contains the essence of the so-called Inverse Ising Problem solution, in which a “Hamiltonian” associated with a given complex system can be inferred from observed statistical correlations among its components. This method is frequently referred to as the Boltzmann machine, since the Boltzmann distribution is present at its core. Generally speaking, the MEM has been applied to systems that can be mapped to Ising-like models, namely, models in which the interacting elements are in an active or inactive state, i.e., a lattice of dipole moments with spins states that are either up or down under the action of an external field and their mutual interactions. For instance, when dealing with neuronal networks, the interactions between neurons reacting to some stimuli are inferred from their firing patterns^15–21^, in which the firing of a spike equate to the spin up state. This strategy has also been successful in characterizing protein–protein interactions^22,23^, the genetic interaction networks from gene expression patterns^24–26^, the collective responses exhibited by flocks of birds^27,28^, and the emergence of collective behavior from the eye movement patterns of a group of people while watching commercial videos^29^ or reading texts^30^.

## Methods

Our initial goal here is to investigate the experimental data reported in Ref.^6^ on the transient synchronization of local neuronal groups, i.e., “spikes”, recorded with microelectrode arrays in neuronal cultures, but from the perspective of a pairwise Maximum-Entropy method. As we show next, we follow the approach proposed in Refs.^16,31–36^ to build an Ising-like model that reproduces the same time-averaged spiking rates and pairwise correlations as the experimental data set, and then evaluate its behaviour in the analogous framework of the corresponding thermal equilibrium properties. Accordingly, let the variable $s_i(t)$ be the binarized time series of spikes of neuron *i* observed at a discretized time $t=1,2,\dots ,M$, such that $s_i = +1$ if it fires and $s_i = -1$ if it does not fire. From these series, we can calculate the time-averaged activity for each neuron *i*,

1 $$\begin{aligned} \left\langle s_{i} \right\rangle ^{obs} = \frac{1}{M}\sum _{t=1}^{M}s_{i}(t), \end{aligned}$$

and the covariances between the spiking sequences of neurons *i* and *j*

2 $$\begin{aligned} C_{ij}^{{ obs}}= \left\langle s_i s_j\right\rangle ^{{ obs}} - \left\langle s_i \right\rangle ^{{ obs}} \left\langle s_j\right\rangle ^{{ obs}}, \end{aligned}$$

where ${\left\langle s_{i} s_{j} \right\rangle = \frac{1}{M}\sum _{t=1}^{M}s_{i}(t)s_{j}(t)}$. Moreover, to model these experimental sequences, we consider that $s_{i}$ corresponds to Ising-like variables on a fully connected network of *N* sites. Therefore, $\left\{ s \right\} = \left\{ s_{1}(t),\dots , s_{N}(t) \right\}$ can describe the system’s state at a given time *t*. The probability distribution $P(\left\{ s \right\} )$ with the smallest number of parameters that represents our system is the one that maximizes the entropy, while reproducing our observations, i.e., $\left\langle s_{i} \right\rangle ^{obs}$ for all *N* neurons and all $N(N-1)/2$ pairs of $C_{ij}^{obs}$. Given these constraints, the form of $P(\left\{ s \right\} )$ is the Boltzmann’s probability distribution,

3 $$\begin{aligned} P(\left\{ s \right\} ) \sim e^{-\mathscr {H}/T}, \end{aligned}$$

where *T* is analogous to a temperature, $\mathscr {H}$ to a Hamiltonian^32^, and we have set ${k_B = 1}$. This distribution corresponds to the least biased representation for an Ising-like system like ours, with known first and second moments. Specifically, as a first approximation, the energy term has the same form of the Ising model,

4 $$\begin{aligned} \mathscr {H} = -\sum _{i=1}^N h_i s_i - \sum _{i > j}^N J_{ij} s_i s_j. \end{aligned}$$

This mathematical correspondence naturally leads us to interpret $h_{i}$ as the action of a local external stimulus on neuron *i*, analogous to a “random field”, and $J_{ij}$ as a “coupling coefficient” between neurons *i* and *j*. Such pairwise couplings or interactions between the neuronal activities give rise to the observed correlations among them. At this point, we compute the local fields $h_{i}$ and the interactions $J_{ij}$ by directly solving the inverse problem given by Eq. (4). For simplicity, here we arbitrarily set the “operating temperature” to $T_{o} = 1$. On their turn, the local fields $h_i$ and interaction constants $J_{ij}$ are obtained through the following iteration scheme:

5 $$\begin{aligned} J_{ij} (n+1)= & {} J_{ij} (n) - \eta (n) \left[ C_{ij}^{MC}-C_{ij}^{{ obs}}\right] , \end{aligned}$$

6 $$\begin{aligned} h_i (n+1)= & {} h_i (n) - \eta (n) \left[ \left\langle s_i \right\rangle ^{MC} - \left\langle s_i \right\rangle ^{{ obs}}\right] , \end{aligned}$$

where *n* is the iteration parameter and we start with $n=1$ and $h_i(n=1)=0$. The covariance $C_{ij}^{MC}$ between two sites *i* and *j* of the Ising network of Eq. (4) is given by $C_{ij}^{MC} = \left\langle s_i s_j \right\rangle ^{MC} - \left\langle s_i \right\rangle ^{MC} \left\langle s_j\right\rangle ^{MC}$, where the statistical average $\left\langle \cdots \right\rangle ^{MC}$ is obtained by performing a Monte Carlo simulation of the model Eq. (4) at temperature $T_{o} = 1$ using $h_i (n)$ and $J_{ij} (n)$. The function $\eta (n)$ is a learning rate which decays like $1/n^{0.4}$^37^. Typically, we iterate till $n = 80{,}000$. Once we infer the values of $h_i$ and $J_{ij}$ that better reproduce the experimentally observed time-averaged activities $\left\langle s_{i} \right\rangle ^{{ obs}}$ and covariances $C_{ij}$, while maximizing the entropy, the Boltzmann probability distribution of Eq. (3) characterizes the statistics of the in vitro datasets.

We analyzed six in vitro samples (called “1” to “6”) consisting of 60 time series of binarized electrode spikes of 1 s divided in 20, 000 time bin each^6^ (see Figs. 1a–c, S1a, S1b, and S1c, for samples 1, 2, 3, 4, 5, and 6, respectively). They were obtained from coronal slices from rat dorsolateral cortex grown at $35.5\,^{\circ }$C for 4–6 weeks before recording, as reported in Ref.^6^.

**Figure 2:**
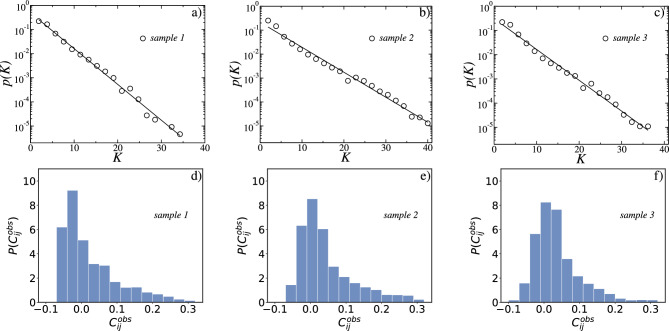
The distribution *P*(*K*) of the measured *K* simultaneous spikes for the in vitro samples 1, 2, and 3 are shown in (**a**–**c**), respectively. The solid black line in each figure is the least-squares fit to the data points of the exponential function $p(K) = \alpha e^{-\beta K}$, with (**a**) $\alpha = 0.50$ and $\beta =0.35$ for sample 1, (**b**) $\alpha = 0.21$ and $\beta = 0.25$ for sample 2, and **(c)** $\alpha =0.31$ and $\beta = 0.30$ for sample 3. The exponential fittings were confirmed by the Kolmogorov–Smirnov (KS) test, yielding a *p* value $> 0.05$ in all cases. In (**d**–**f**) are the distributions of correlation coefficients $C_{ij}^{obs}$ between pairs of electrodes for the in vitro samples 1, 2 and 3, respectively.

## Results and discussion

### In vitro sample characterization

In Fig. 2a–c we show the probability *P*(*K*) that *K* electrodes fire simultaneously for in vitro sample 1, 2, and 3, respectively. The data follow an exponential decay $P(K) = \alpha e^{-\beta K}$, whose parameters depend on the sample considered. From the least-squares fit to the data points we obtained $\alpha = 0.50$ and $\beta =0.35$ for sample 1, $\alpha = 0.21$ and $\beta = 0.25$ for sample 2, and $\alpha =0.31$ and $\beta = 0.30$ for sample 3. The exponential fittings were confirmed by the Kolmogorov–Smirnov (KS) test, yielding a *p* value $> 0.05$ in all cases. Therefore, the data exhibit a Poisson-like distribution, which would suggest uncorrelated events. We calculate next for every pair of sites $\{ij\}$ the corresponding time-averaged correlation $C_{ij}^{{ obs}}$. Their distributions are shown in Fig. 2d–f for the in vitro samples 1, 2, and 3, respectively. All three in vitro samples have their maximum around zero, but with skewness towards positive values. Moreover, the in vitro distributions are qualitatively different from each other. The question that naturally arises is how this difference will be expressed by the Ising-like models that we construct next.

**Figure 3:**
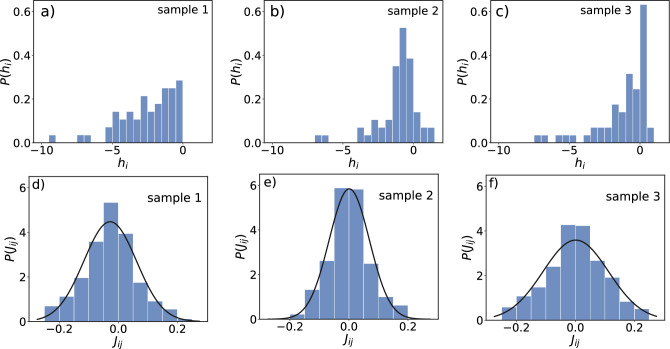
Figures (**a**–**c**) show the distributions of local fields $h_{i}$ after learning for in vitro samples 1, 2, and 3, respectively. All distributions display skewness towards negative values. Figures (**d**–**f**) show the distributions of the coupling coefficients $J_{ij}$ between $N = 60$ electrodes for in vitro samples. The black continuous lines in (**d**–**f**) correspond to a Gaussian fitting and the adequacy of this model was confirmed by the Kolmogorov–Smirnov (KS) test.

### Distributions of the learned parameters of the model in Eq. (4)

In order to apply the Boltzmann machine, we solved Eqs. (5) and (6) simultaneously to calculate for each sample the local fields $h_i$ and the coupling constants $J_{ij}$. Their distributions are shown in Fig. 3. We see that, for all samples, the fields $h_i$ are mostly negative but the most likely values depend on the sample considered. For samples 1 (Fig. 3a) and 2 (Fig. 3b) these are close to zero on the negative side while for sample 3 (Fig. 3c) the most likely value is close to zero on the negative side. Furthermore, all distributions exhibit a skewness towards negative fields. Since our system has only 60 electrodes, the sample to sample variations are considerable. As also shown in Fig. 3d–f the distributions of the interaction constants $J_{ij}$ are in all cases symmetrically centered around zero. Moreover, from the Kolmogorov–Smirnov (KS) test, Gaussian fittings appropriately describe the data for all samples.

**Figure 4:**
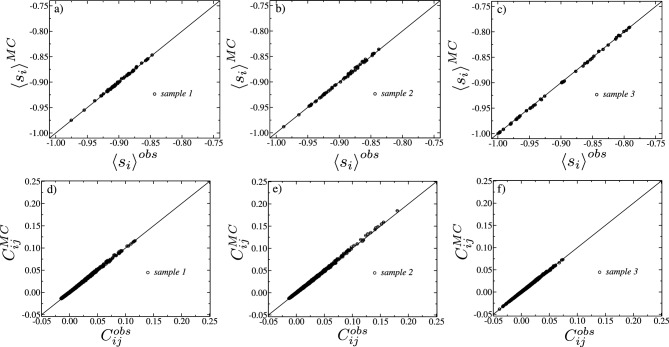
In (**a**–**c**) are shown the magnetizations $\left\langle s_{i} \right\rangle ^{MC}$ versus $\left\langle s_{i} \right\rangle ^{obs}$ for the in vitro samples 1, 2, and 3, respectively. The same in (**d**–**f**), but for the correlations $C_{ij}^{MC}$ versus $C_{ij}^{obs}$ obtained from the in vitro samples 1, 2, and 3, respectively. The error bars represent the standard deviations calculated from $10^{5}$ samples generated through repeated Monte Carlo runs. The solid black lines correspond to the function $y=x$.

**Figure 5:**
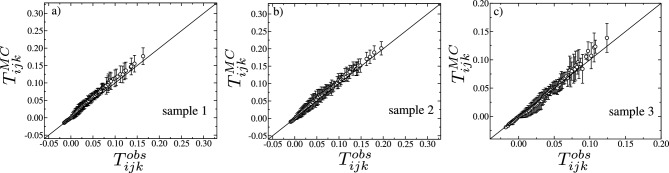
Test to verify if the three-point correlations $T_{ijk}^{MC}$ from the Monte Carlo simulations of the Ising-like model of Eq. (4) are able to reproduce the three-point correlations $T_{ijk}^{obs}$ of the experimental in vitro samples 1, 2 and 3, in (**a**–**c**), respectively. The solid black lines correspond to the function $y=x$. The triplets are binned into 100 populated bins and the error bars are the standard deviations across the bins.

### Test to verify how well the Ising-like model of Eq. (4) can reproduce the measured time averages

To verify how well the Ising-like model Eq. (4) is able to reproduce the measured time averages of the experimental data, we show in Fig. 4a–c $\left\langle s_{i} \right\rangle ^{MC}$ against $\left\langle s_{i} \right\rangle ^{obs}$ for every site *i* of the in vitro sample 1, 2, and 3, respectively, and in Figs. S2a, S2b, and S2c for the samples 4, 5 and 6, respectively. The same is shown in Fig. 4d–f but for the two-point correlations $C_{ij}^{MC}$ against $C_{ij}^{obs}$ for every pair of sites *i* and *j* of the samples 1, 2, and 3, respectively, and in Figs. S3a, S3b, and S3c for the samples 4, 5 and 6, respectively. Clearly, the agreement is excellent in all cases. For samples 1, 2, and 3, as illustrated in Fig.  5a–c, respectively, the model demonstrates remarkable accuracy in predicting the experimentally observed correlations among triplets of neurons. The precision of these predictions is quantitatively supported by the relative errors, $\left\langle (T_{ijk}^{MC}-T_{ijk}^{obs})/T_{ijk}^{obs}\right\rangle$, which amount to $0.095\%$, $0.091\%$, and $0.120\%$ for samples 1, 2, and 3, respectively. These deviations are unusually smaller than those observed in previous studies^16^, highlighting the adequacy of the Ising-like model to describe the time-averaged spiking behavior of these in vitro neuronal networks. Moreover, since the three-point correlations were not explicitly utilized in the correlation function matching procedure, these findings strongly indicate that the machine demonstrates a robust level of generalization for these samples, providing us with a solid basis to confidently infer thermodynamic properties from the learned parameters. In the case of samples 4, 5, and 6, as depicted in Figs. S4a, S4b, and S4c, respectively, the models accurately reflect the rankings of the triplets, in the sense that triplets observed experimentally to be more strongly correlated are also predicted to be relatively more strongly correlated within the set of model predictions. Nonetheless, the models’ inability to quantitatively replicate the observed three-point correlations prevents our examination of their thermodynamic properties.

### Critical properties of the Ising-like model

After showing that the Ising-like model describes to a satisfactory degree the time-averaged properties of our experimental samples, we now proceed with a more detailed investigation of its properties in terms of the implicit thermodynamic analogy brought about naturally^33^. The model adopted here is in fact an extension of the famous Sherrington-Kirkpatrick (SK) spin glass^12^ without averaging over quenched disorder, but including a random field. Since the coupling constants of our model are distributed around zero, we expect frustration effects and, due to the long-range interactions, the occurrence of mean-field behaviour. In addition to the SK spin glass, however, we have a predominantly negative quenched random field. This extension of the SK model was in fact studied by Hadjiagapiou^13^ for the case in which both the interaction constants and the local fields are Gaussian distributed. We assume that, despite some differences in the distributions, our model should have a similar phase diagram as in Ref.^13^, namely, as a function of temperature, either a spin glass to paramagnetic transition for weak negative fields (below the de Almeida–Thouless (AT) line^14^) or a ferromagnetic-paramagnetic phase transition for stronger negative fields (above the AT line). Moreover, the upper critical dimension should be that of the random field Ising model^38^.

**Figure 6:**
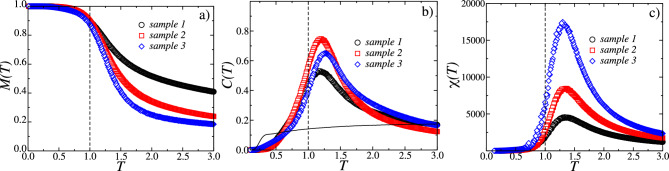
Thermodynamic analysis for the in vitro samples 1, 2, and 3. In (**a**) is shown the magnetization *M*(*T*) as function of temperature *T*. In (**b**) is shown the specific heat *C*(*T*) as a function of temperature *T*. We also show the specific heat for sample 1 after reshuffling the data (solid black line). In (**c**) is shown the susceptibility $\chi (T)$ against temperature *T*. In all cases, the dashed line corresponds to the operation temperature $T_{o} = 1$.

Under this analogous thermodynamic framework, for every spin configuration $\{s_i\}$, one can define its magnetization ${M(T)=(1/N)\sum _{i}s_i}$ and its energy ${E(T)=(1/N)\sum _{ij}J_{ij}s_i s_j + (1/N)\sum _{i}{h_i s_i}}$. The order parameter for a ferromagnetic phase transition is given by the average magnetization $\left\langle M(T)\right\rangle ^{MC}$, and the fluctuation-dissipation theorem provides the following expression for the specific heat:

7 $$\begin{aligned} C (T) = \frac{1}{T^{2}} \left( \left\langle E(T)^2\right\rangle ^{MC} - \left( \langle E(T)\right\rangle ^{MC})^2\right) , \end{aligned}$$

and the susceptibility

8 $$\begin{aligned} \chi (T) = \frac{1}{T}\left( \left\langle M(T)^2\right\rangle ^{MC} - \left( \langle M(T) \right\rangle ^{MC})^2\right) . \end{aligned}$$

In Fig. 6a the ferromagnetic order parameter $\langle M(T)\rangle ^{MC}$ is plotted against temperature *T* for the three in vitro samples. We have verified that these curves do not depend on the initial configuration used in the Monte Carlo simulation. The fact that we do not observe hysteresis indicates that the transition might be second order and not first order as observed, for example, in retinal ganglion cell spike trains^21^. Figure 6b shows the specific heat *C*(*T*) as a function of temperature for the three in vitro samples. The curves attain maxima at a temperature $T_{c}^{*}$ that depends on the sample considered, with $T_{c}^{*} = 1.18$ for sample 1, $T_{c}^{*} = 1.20$ for sample 2, and $T_{c}^{*} = 1.27$ for sample 3. Also shown in Fig. 6b is the dependence of the specific heat on temperature obtained by randomly shuffling the spikes in the time series of the in vitro sample 1, i.e., by suppressing the intrinsic correlations present in the spike sequence. We see that, in this case, the sharp maximum of the specific heat is dramatically attenuated, which means that there is no ferromagnetic phase transition any more as a function of temperature. Possibly, this case falls below the AT line and might exhibit a spin glass phase at low temperatures. Higher order correlations seem important for neurons that are close to each other^36^. In order to take those into account, the method can be generalized to multi-spin interactions^35^. We also studied the susceptibilities $\chi (T)$ of the Ising-like models for samples 1,2, and 3, shown in Fig. 6c. They also exhibit a maximum at a given temperature $T_{c}^{*}$, but unlike specific heat curves, the maxima of the susceptibility occur at the same value $T_{c}^{*} = 1.34$. Furthermore, the maxima of the susceptibility $\chi _{\max }$ are quite different between the samples, despite having the same number of spins, with $\chi _{\max }^{(3)}> \chi _{\max }^{(2)} > \chi _{\max }^{(1)}$ for sample 3, 2, and 1, respectively.

## Conclusion

In summary, we analyzed time-averaged experimental data from the activities of in vitro neuronal networks. By implementing a Pairwise Maximum-Entropy method alongside an inverse binary Ising-like model, we successfully identified the local fields and interaction couplings that best represent the average neuronal activities and the statistical correlations between neuronal pairs. This approach confirmed the model’s effectiveness in capturing the inherent complexities of neuronal network dynamics, where specific information about neuron types is reflected in the local fields, and a symmetric distribution in interaction constants emerges as a characteristic trait of the network’s interaction framework.

The investigation into the thermodynamic properties of these models revealed typical features of second-order phase transitions between ferromagnetic and paramagnetic states at temperatures $T_{c}^{*}$ above, but not far from unity, which corresponds to the operating temperature, $T_{o}=1$, utilized in the Maximum-Entropy method. This finding extends the thermodynamic analogy within our Ising-like models, giving support to the hypothesis of criticality in neuronal network behavior. It points out the relevance of critical phenomena in understanding the functionality and complexity of neuronal networks.

The exceptional alignment between our model predictions and experimental observations, particularly in the accurate rendering of three-point correlations, demonstrates a case of success of the methodology. This precision emphasizes the Ising-like model’s adequacy in representing the nuanced spiking behavior of neuronal networks, surpassing previous modeling attempts in terms of accuracy and reliability. These findings not only validate the criticality hypothesis in neuronal networks, but also highlight the potential of using thermodynamic and statistical physics concepts to explore neural computation and information processing.

### Supplementary Information

Supplementary Information.

## Data Availability

The source data for all figures in this study are provided for this paper. Source data are provided with this paper. The in vitro data is available at https://github.com/cesampaiof/Ising-like-model-neuronal-networks.
