# Supplementary material for: Ising-like model replicating time-averaged spiking behaviour of in vitro neuronal networks
Source: Sci Rep. 2024 Mar 25;14:7002. doi: 10.1038/s41598-024-55922-9 (PMC11319664; doi:10.1038/s41598-024-55922-9)
Supplement: Supplementary file 1 — Supplementary Information. [file 41598_2024_55922_MOESM1_ESM.pdf]

Supplementary information: Ising-like model replicating time-averaged spiking behaviour of in vitro neuronal networks

Cesar I.N. Sampaio Filho[1], Lucilla de Arcangelis[2], Hans J. Herrmann[1,3],
Dietmar Plenz[4], Patrick Kells[4], Tiago Lins Ribeiro[4], and José S. Andrade Jr.[1]

[1]*Departamento de Física, Universidade Federal do Ceará, 60451-970 Fortaleza, Brazil*

[2]*Department of Mathematics and Physics,*
University of Campania "Luigi Vanvitelli", 81100 Caserta, Italy

[3]*PMMH, ESPCI, CNRS UMR 7636,*
7 quai St. Bernard, 75005 Paris, France

[4]*Section on Critical Brain Dynamics,*
NIH, Bethesda, Maryland 20892, USA

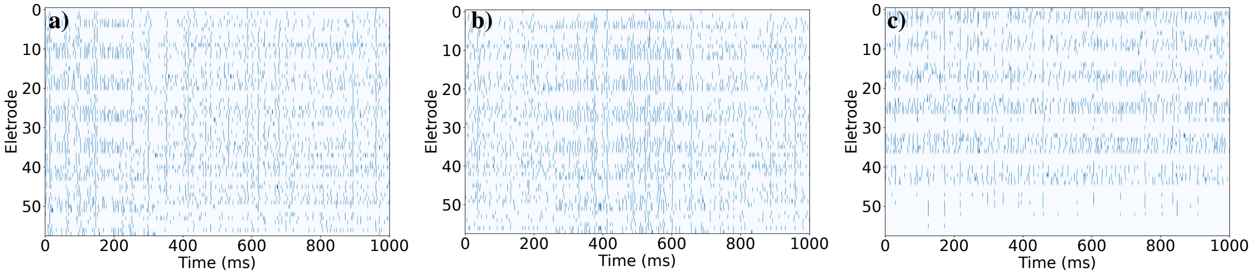

Fig. S1 The ensemble of investigated time series of binarized spikes spanning a total of 1 sec for 60 electrodes of the in vitro samples 4, 5 and 6 are shown in (a), (b) and (c), respectively. Each horizontal line represents the temporal spiking series of a local group at an electrode or single neuron, respectively, whereas each vertical line represents the state of the system at time t. A blue point corresponds to a spike (+1) and an empty place to no spike (−1).

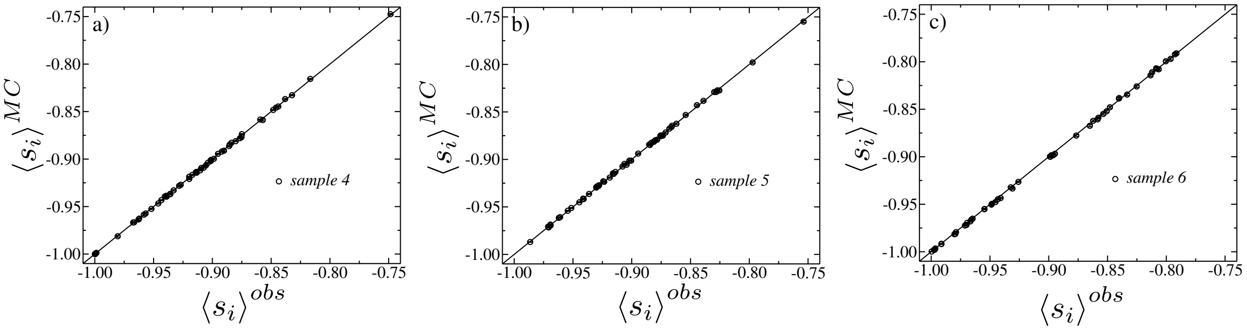

Fig. S2 In (a), (b), and (c) are shown the the magnetizations $\langle s_i \rangle^{MC}$ vs $\langle s_i \rangle^{obs}$ for the in vitro samples 4, 5, and 6, respectively. The error bars represent the standard deviations calculated from 10^5 samples generated through repeated Monte Carlo runs. The solid black lines correspond to the function $y = x$.

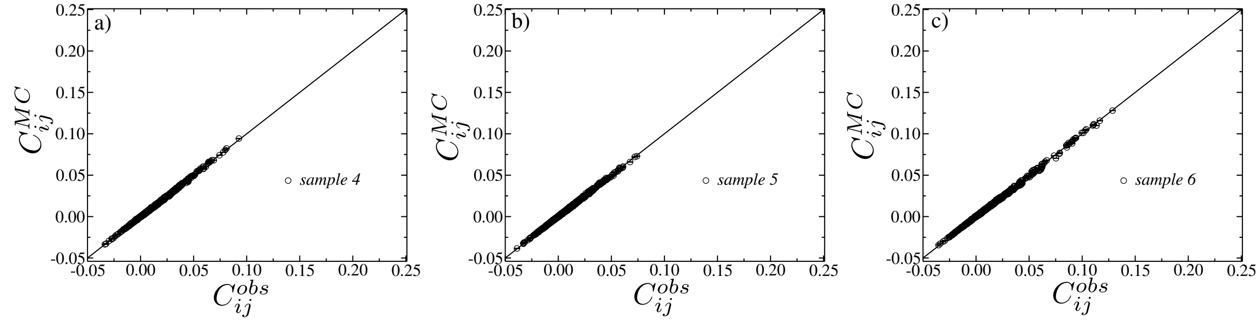

Fig. S3 In (a), (b), and (c) are shown the the correlations C_{ij}^{MC} vs C_{ij}^{obs} for the in vitro samples 4, 5, and 6, respectively. The error bars represent the standard deviations calculated from 10^5 samples generated through repeated Monte Carlo runs. The solid black lines correspond to the function $y = x$.

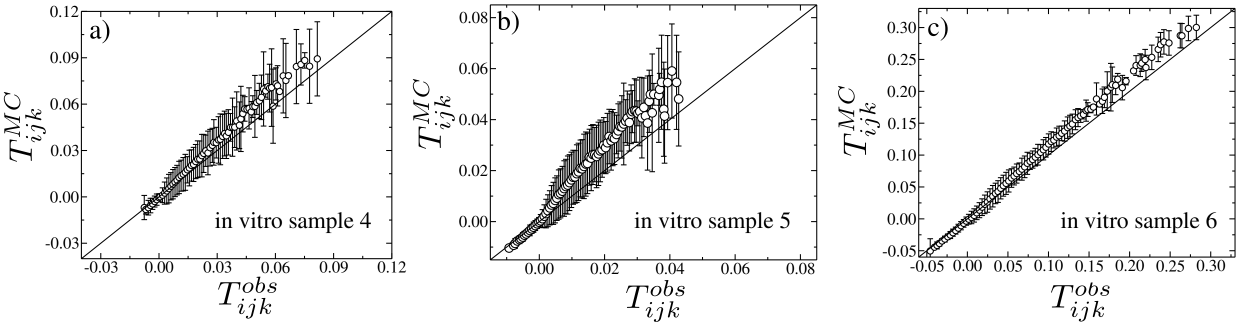

Fig. S4 Test to verify if the three-point correlations T_{ijk}^{MC} from the Monte Carlo simulations of the Ising-like model of Eq. (4) are able to reproduce the three-point correlations T_{ijk}^{obs} of the experimental in vitro samples 4, 5 and 6, in (a), (b) and (c), respectively. The solid black lines correspond to the function $y = x$. The triplets are binned into 100 populated bins and the error bars are the standard deviations across the bins.
